# Subjective prosthodontic treatment need, tooth loss and associated factors among dental patients in Dar es salaam, Tanzania

**DOI:** 10.4314/ahs.v21i4.49

**Published:** 2021-12

**Authors:** Lorna C Carneiro, Silas Sembiko, Joyce R Masalu

**Affiliations:** 1 Department of Restorative Dentistry, School of Dentistry, Muhimbili University of Health and Allied Sciences, P. O. Box 65451, Dar es salaam, Tanzania; 2 Department of Orthodontic, Pedodontic and Community Dentistry, School of Dentistry, Muhimbili University of Health and Allied Sciences, P. O. Box 65014, Dar es salaam, Tanzania

**Keywords:** Subjective prosthodontic treatment need, tooth loss, dental patients, Dar es salaam, Tanzania

## Abstract

**Background:**

A full complement of teeth has been shown to be a prerequisite for a healthy masticatory system and satisfactory function and having tooth loss can be undesirable.

**Objective:**

To determine the subjective prosthodontic treatment need, tooth loss and associated factors among patients attending dental clinics in Dar-es-salaam, Tanzania.

**Methods:**

This cross-sectional study was conducted among subjects with tooth loss aged 18 years and above attending public dental clinics in Dar-es-Salaam, Tanzania. A questionnaire and clinical examination were used to obtain data. Chi-Square test and logistic regression analyses were performed and a p-value of ≤0.05 was considered to be statistically significant.

**Results:**

Majority of the 402 participants were aged 18–34 years (52.2%), females (64.9%) and with primary level of education or less (52.2%). Subjective prosthodontic treatment need was indicated by more than half of the participants 54.2%) and those who lost 4 or more teeth and those who lost teeth upper anterior had higher odds of expressing subjective treatment need (OR=2.6; CI=1.5–4.3 & OR=4.9; CI=2.2–10.8 respectively).

**Conclusions:**

This study highlights that having four or more missing teeth and having tooth loss in the anterior location of the upper jaw were significant contributing factors in expressing patient's subjective prosthodontic treatment need.

## Introduction

A full complement of teeth is a prerequisite for a healthy masticatory system, satisfactory function^1^ and aesthetics^2^. Having tooth loss is undesirable irrespective of the cause^3^ and tooth retention profile of populations is influenced not only by dental diseases but also by socio-economic, behavioral and attitudinal characteristics^4^.

Subjective prosthodontic treatment need is determined by functional, esthetic, psychological and social impacts due to tooth loss^5^. Different levels of prosthodontic interventions are needed as a standard of care to improve conditions of persons with tooth loss^6^. Nevertheless not all patients with incomplete dental arch need treatment or perceive their condition as harmful or deleterious^7^.

In Tanzania, tooth loss is a common finding^8^ with reported objective need for tooth replacement being higher among patients having missing teeth in both anterior and posterior regions^9^. However, there is paucity of information on the subjective prosthodontic treatment need of patients in relation to number and location of missing teeth. This study therefore aimed to determine the subjective prosthodontic treatment need, tooth loss and associated factors among patients attending public dental clinics in Dar es Salaam, Tanzania. The results of this study will provide baseline information for comparative studies regarding the subjective prosthodontic treatment need of Tanzanians, the pattern of tooth loss and associated factors. Furthermore, it will assist in the planning of preventive strategies and objective need for prosthodontic treatment.

## Methods

This cross-sectional hospital-based study involved dental patients aged 18 years old and above who attended public dental clinics in Dar es Salaam, Tanzania. Dar es salaam was purposively selected due to the population density from all over Tanzania^10^ and because of the likelihood of getting many patients in the clinics. An urban and rural representation was obtained by selection of one public dental clinic from each of the three municipalities (Kinondoni, Ilala and Temeke) in Dar es salaam. The consultant referral dental clinic at the Muhimbili National Hospital was further selected as it receives referred patients from all over the country. At each hospital, dental patients aged 18 years old and above who attended the selected dental clinics during the study period were conveniently selected so as to obtain the estimated sample size of 422.

Enrolled in the study were attending patients aged 18 years and above with one or more missing teeth. Subjects excluded from the study were patients who did not have tooth loss, those mentally challenged/handicap, presenting with emergency dental conditions or oral tumors/cancers and those with removable or fixed prosthesis.

Ethical clearance was granted by the Muhimbili University of Health and Allied Sciences (MUHAS) while permission to conduct the research in respective dental clinics was obtained from the respective administrative authorities. Prior to obtaining individual written consent patients awaiting dental treatment at each clinic were informed of the study objectives and assured of their confidentiality and the right to participate or withdraw from the study without penalty.

Data were collected from the study participants through an interview using a structured questionnaire followed by a clinical examination. Following consent subjects were seated comfortably in the dental chair and interviewed by one of the investigators (SS) using a structured questionnaire. The first section of the questionnaire obtained information with regards to socio demographic characteristics. Participants were categorized by age into two groups 18–34 years = 0 and 35–85 years =1 based on the median age of 34 years while their sex was recorded as male = 0 or female = 1. The level of education of participants was recorded as having “no formal education, primary education, secondary education and college/university education” which were then dichotomized into primary education or less = 0 and secondary education or more = 1.

The second section of the questionnaire determined if a subject had missing teeth based on no =0 and yes =1 response and those with missing teeth reported their subjective prosthodontic treatment need using a yes = 0 or no =1 response.

Clinical examination of subject's oral cavity was then performed by the same investigator using a mouth mirror and illumination from the overhead light. Findings were recorded in a clinical survey form that had a chart of all the 32 teeth. Teeth that were present in the oral cavity and with more than half of its crown intact were scored = 0 while teeth that were missing or with more than half of its crown absent were scored =1.

Subjects were further grouped according to number of missing teeth (1–3=0 and 4 or more =1) and location of missing tooth/teeth (tooth number 13–23 = upper anterior jaw; tooth number 14–18 or 24–28 = upper posterior jaw; tooth number 33–43 = lower anterior jaw and tooth 34–38 or 44–48 = lower posterior jaw). Intra-examiner consistency was assessed daily by randomly selecting 10% of patients enrolled in the study. Kappa value of 0.87 was obtained.

To assess for clarity and to ensure that the questionaire measured what it was supposed to measure and that the clinical record form was usable, a field testing was conducted on 40 conveniently selected patients attending the dental clinic of Muhimbili National Hospital. Test-retest for assessing reliability of the questionnaire was not done because it was not possible to invite participants for a second administration of the questionnaire. Calibration of examiner SS with respect to clinical assessment was carried out against LCC.

The data were processed and analyzed using SPSS software for Windows version 20. Cross-tabulations and Chi-Square test was used to determine bivariate associations with p-value ≤ 0.05 being considered to be statistically significant. Multiple logistic regression of dependent variable and independent variables was performed to control for confounding effects.

## Results

A total of 402 dental patients with one or more missing teeth participated in the study giving a response rate of 95.3%. Age ranged from 18–85 years with mean age of 36.16 ±13.246 years and median age of 34 years. The majority of subjects were from age group 18–34 years (52.2%), females (64.9%) and having primary or less level of education (52.2%). Many more participants had three or less missing teeth (56.5%) with many more missing teeth in the lower posterior jaw (84.6%), upper posterior jaw (66.4%), upper anterior jaw (16.7%) and lower anterior jaw (4.5%) respectively. Subjective prosthodontic treatment need was expressed by 54.2% of the participants (Table 1).

**Table 1:** Distribution of participants by sociodemographic factors, missing teeth and subjective treatment need, (N=402)

Variables		n	%
*Age Group (years)*	18–34	210	52.2
	35–85	192	47.8
*Sex*	Male	141	35.1
	Female	261	64.9
*Level of Education*	Primary School or less	210	52.2
	Secondary School or more	192	47.8
*Number of missing teeth*	1–3	227	56.5
	4 or more	175	43.5
*Location of missing teeth*	Upper jaw - Anterior	67	16.7
	Upper jaw - Posterior	267	66.4
	Lower jaw - Anterior	18	4.5
	Lower jaw - Posterior	340	84.6
*Subjective treatment* *need*	No	184	45.8
	Yes	218	54.2

Table 2 shows the distribution of number and location of missing teeth of participants by socio-demographic characteristics. A statistically significant higher proportion of those who were in age group 35–85 years (67.4%), females (70.3%) and those with primary education or less (60.0%) had 4 or more missing teeth in comparison to their counterparts. In regards to location of missing teeth, a significantly higher proportion of participants in 35–85 years had missing teeth in the anterior area of the upper jaw (74.6%) with no significant differences observed between males and females or between the different levels of education. Having more missing teeth in the posterior area of the upper jaw was significantly linked to being of age group 35–85 years age group (56.9%) and having primary or less education (56.9%). There was no significant association between different sexes and missing teeth in upper posterior location. In the lower jaw, the observed statistically significant difference between those aged 35 and above who had missing teeth in the anterior area as compared to none in age less than 35 years is noted. A significantly higher number of those aged 35 and above who had missing teeth in the anterior area were having primary or less education (88.9%). Having missing teeth in the posterior area of lower jaw showed no association with age, sex or level of education.

**Table 2:** Distribution of participants by missing teeth and socio-demographic characteristics

Demographic characteristics							
	Age Group (years)		Sex		Level of education		Total
Missing Teeth	18–34	35–85	Male	Female	Primary or less	Secondary or more	
*Number of missing teeth*							
3 or less	153 (67.4)	74 (32.6)	89 (39.2)	138 (60.8)	105 (46.3)	122 (53.7)	227
4 or more	57 (32.6)	118 (67.4)***	52 (29.7)	123 (70.3)*	105 (60.0) **	70 (40.0)	175
*Location of missing teeth*							
Upper jaw - anterior							
No	193 (57.6)	142 (42.4)	116 (34.6)	219 (65.4)	172 (51.3)	163 (48.7)	335
Yes	17 (25.4)	50 (74.6)***	25 (37.3)	42 (62.7)	38 (56.7)	29 (43.3)	67
Upper jaw - posterior							
No	95 (70.4)	40 (29.6)	53 (39.3)	82 (60.7)	58 (43.0)	77 (57.0)	135
Yes	115 (43.1)	152 (56.9)***	88 (33.0)	179 (67.0)	152 (56.9) **	115 (43.1)	267
Lower jaw - anterior							
No	210 (54.7)	174 (45.3)	134 (34.9)	250 (65.1)	194 (50.5)	190 (49.5)	348
Yes	0 (0.0)	18 (100)***	7 (38.9)	11 (61.1)	16 (88.9) ***	2 (11.1)	18
Lower jaw - posterior							
No	33 (53.2)	29 (46.8)	26 (41.9)	36 (58.1)	26 (41.9)	36 (58.1)	62
Yes	177 (52.1)	163 (47.9)	115 (33.8)	225 (66.2)	184 (54.1)	156 (45.9)	380

p≤ 0.001***, p≤0.01**, p≤0.05*

The distribution of subjective prosthodontic treatment need of participants by socio-demographic characteristics is shown in Table 3. Statistically significantly higher proportion (61.5%) of participants aged 35–85 years expressed subjective prosthodontic treatment need in comparison to those aged 18–34 years (47.6%). The subjective prosthodontic treatment need of participants did not vary across sexes or education levels.

**Table 3:** Distribution of subjective prosthodontic treatment need of participants by sociodemographic characteristics

		Subjective prosthodontic treatment need		
Socio demographic characteristics		No	Yes	Total
*Age (years)*	18–34	110 (52.4)	100 (47.6)	210**
	35–85	74 (38.5)	118 (61.5)	192
*Sex*	Male	66 (46.8)	75 (53.2)	141^ns^
	Female	118 (45.2)	143 (54.8)	261
*Level of* *education*	Primary or less	100 (47.6)	110 (52.4)	210 ^ns^
	Secondary or more	84 (43.8)	108 (56.2)	192

^ns^ =Not significant; p≤0.01**

Shown in Table 4 is the distribution of subjective prosthodontic treatment need of participants by number and location of missing teeth. The subjective prosthodontic treatment need of participants who were missing 4 or more teeth (70.3%) was statistically significantly higher than those who were missing 3 or less (41.9%). The subjective prosthodontic treatment need was statistically significantly higher among participants with missing teeth in the anterior location of the upper jaw (86.6%), posterior location of the upper jaw (58.4%) and anterior location of lower jaw (77.8%). There was no statistically significant difference in subjective treatment need between participants having or not having missing teeth in the posterior location of lower jaw.

**Table 4:** Distribution of subjective prosthodontic treatment need of participants by number and location of missing teeth

		Subjective prosthodontic treatment need		
Missing teeth		No	Yes	Total
*Number of missing teeth*				
	3 or less	132 (58.1)	95 (41.9)	227**
	4 or more	52 (29.7)	123 (70.3)	175
*Location of missing teeth*				
Upper jaw -anterior	No	175 (52.2)	160 (47.8)	335**
	Yes	9 (13.4)	58 (86.6)	67
Upper jaw -posterior	No	73 (54.1)	62 (45.9)	135*
	Yes	111 (41.6)	156 (58.4)	267
Lower jaw -anterior	No	180 (46.9)	204 (53.1)	384*
	Yes	4 (22.2)	14 (77.8)	18
Lower jaw -posterior	No	30 (48.4)	32 (51.6)	62^ns^
	Yes	154 (45.3)	186 (54.7)	340

^ns^ =Not significant; p≤0.01**, p≤ 0.05*

Table 5 shows adjusted odds ratios (ORs) and 95% confidence intervals (CIs). To determine the likelihood of expressing subjective prosthodontic need; independent variables including age, number of missing upper anterior, upper posterior and lower anterior location teeth were regressed upon subjective prosthodontic treatment need as a dependent variable.

**Table 5:** Odds ratios (ORs) and 95% confidence intervals (CIs) for subjective prosthodontic treatment need according to age, number of missing teeth, teeth missing in upper anterior, upper posterior and lower anterior location

	p-value	OR	95% CI
Age			
18–34yrs vs. ≥35 yrs	0.711	1.090	0.692 – 1. 716
Number of missing teeth			
≤3 teeth vs. ≥4 teeth	0.001	2.613	1.557 – 4.386
Teeth missing in upper jaw -anterior area			
Yes vs. No	0.001	4.968	2.272 – 10.862
Teeth missing in upper jaw - posterior area			
Yes vs. No	0.607	0.874	0.523 – 1.460
Teeth missing in lower jaw - anterior area			
Yes vs. No	0.918	0.932	0.247 – 3.514

Those with 4 or more missing teeth (OR=2.6; CI=1.56–4.39) and those with missing teeth in the upper anterior location (OR=4.97; CI=2.27–10.86) had higher odds of expressing subjective prosthodontic treatment need. Nagelkerke R2=0.172≈17%.

## Discussion

This hospital based cross-sectional study assessed the subjective prosthodontic treatment need of patients aged 18–85 years attending selected public dental clinics in Dar es Salaam, Tanzania. The involvement of public dental clinics was based on expectation of getting a wide coverage of Tanzanians with missing teeth from both urban and rural populations. Limitation of the sampling methods used and possible recall bias with regards to the questionaire were considered. However, the results provide an insight on the expressed subjective prosthodontic treatment need of Tanzanian patients and the influence of socio demographic characteristics, number and location of missing teeth. The data obtained from this study can be used for comparison with other studies within and outside the country.

This study had a higher number of subjects within the age group of 35–72 years with 4 or more missing teeth than their younger counterparts. Similar findings were reported in studies done in Asia 11, Brazil 12 and Europe^13^. On the contrary, a study done in Uganda^14^ reported young adults to have lost more teeth. The higher number of missing teeth amongst the older age group in this study could be related to inadequate oral hygiene practices and possibly the cumulative effect of oral diseases^15^. In addition barriers to seeking oral health care experienced by many in the past could have led to emergency oral care services in form of tooth extraction 16. Unlike other studies 17 where males had more missing teeth, this study observed a higher number of missing teeth in females. Similar finding have been reported in another study done in Tanzania 8 and other studies in Uganda 14, Sudan 18 and Nigeria 19. Higher number of missing teeth reported among females in this study could be attributed to their behavior of snacking and that of visiting the dentist more frequently 20.

The higher number of missing teeth amongst those with lower level of education in this study was similarly reported in other studies^21^,^22^. Contrastingly a study done in Nigeria showed no association between number of teeth lost and education level 17. Participants with lower level of education in this study may have not received adequate oral health knowledge on preventive behaviors. Furthermore, utilization of oral health services among those who are less educated in most instances is for emergency oral health care rather than restorative care^23^.

In agreement with other studies done in Nigeria^17^ and Brazil 24 this study also observed that missing teeth in the upper and lower anterior locations was associated with the 35–85 years age group. Observed findings from this study could be due to financial constraints faced by older adults who tend to seek care on a problem-oriented basis 25.

Missing teeth in the posterior location of the upper jaw was also common among the older age group in Nigeria^17^. Other studies done in Tanzania 26,27 and Kenya^28^ found no association between missing teeth in the posterior location of upper jaw and age. The observed findings could be related to the differences in sampling of subjects, however, it could also be related to the tooth anatomy of posterior teeth which predisposes to dental caries.

In this study missing teeth in the upper posterior location and lower anterior location was associated with having primary education or less and these findings corroborate with studies done in Thailand 29, Japan^30^ and Brazil^31^. It is possiblthat people with lower level of education lack awareness on prevention of oral diseases and available treatment options.

Although a study done in Nigeria reported that males lost more anterior teeth while females lost more posterior teeth^17^ this study showed no association between location of missing teeth and sex. The possible reason for the lack of association between location of missing teeth and sex could be related to provision of emergency treatment care to all in need regardless of sex.

The results from this study were in agreement with a number of studies^6^,^32^, whereby the subjective prosthodontic treatment need was associated with having an age of 35 or more years, however, a study done in Norway reported no association between subjective prosthodontic treatment need and age 33. The cumulative effects of dental diseases can explain the subjective treatment need amongst the older age group.

Unlike a study done in Norway 34 that reported a higher subjective prosthodontic treatment need in males, a study done in Poland 35 reported a higher subjective prosthodontic treatment need in females. These findings were contrary to findings reported in this study that showed no association between subjective prosthodontic treatment need and sex. Chewing disability due to missing teeth experienced by both sexes 12 could explain the lack of difference observed in subjective prosthodontic treatment need between sexes.

While a study done in Brazil reported association between subjective prosthodontic treatment need with higher level of education 36, this study showed no association between subjective prosthodontic treatment need and education level. It is possible that the subjective prosthetic treatment need of an individual is not influenced by the level of education but rather satisfaction with oral function and aesthetics.

Contrasting to findings from a study in Saudi Arabia^5^ the subjective prosthodontic treatment need of participants in this study who were missing 4 or more teeth was high. The higher subjective prosthodontic treatment need among participants in this study with four or more missing teeth could be related to their reduced oral functionality^37^.

Although it has been reported that the demand for prosthetic replacement is associated with position of the missing teeth^38^ this study reported a higher subjective prosthetic treatment need among participants with missing teeth in the anterior area of the upper jaw, anterior area of the lower jaw and posterior area of the upper jaw. Wide smiles that compromise aesthetics from visibility of edentulous gaps in the posterior area of the upper jaw could have been a contributing factor.

Similar to findings from a study done in Italy 39, the subjective prosthodontic treatment need of participants in this study was significantly associated with 4 or more missing teeth. The observed subjective prosthodontic treatment need can be due to affected aesthetics and oral function 40.

Association between subjective prosthodontic treatment need and loss of teeth in the upper anterior location was also reported in Poland 41. This observed subjective prosthodontic treatment need can be explained by social considerations, compromised aesthetics, speech and/or decreased self-esteem 42.

## Conclusion

This study highlights that having four or more missing teeth and having tooth loss in the anterior location of the upper jaw were significant contributing factors in expressing patient's subjective prosthodontic treatment need.
